# Women Veterans Experience with the VA MOVE! Weight Management Program

**DOI:** 10.1089/whr.2019.0009

**Published:** 2020-03-04

**Authors:** Bryan C. Batch, Candace S. Brown, Karen M. Goldstein, Susanne Danus, Nina R. Sperber, Hayden B. Bosworth

**Affiliations:** ^1^ Division of Endocrinology, Department of Medicine, Durham VA Medical Center, Durham, North Carolina.; ^2^ Division of Endocrinology, Metabolism and Nutrition, Duke University, Durham, North Carolina.; ^3^ Department of Public Health Sciences, UNC-Charlotte, North Carolina.; ^4^ Motivated Cognition and Aging Brain Laboratory, Center for Cognitive Neuroscience, Duke University, Durham, North Carolina.; ^5^ Durham VA Health Care System, Durham Center of Innovation to Accelerate Discovery and Practice Transformation, Durham, North Carolina.; ^6^ Department of Population Health Sciences, Duke University School of Medicine, Durham, North Carolina.; ^7^ Department of Psychiatry and Behavioral Sciences, School of Nursing, Duke University, Durham, North Carolina.

**Keywords:** nutrition, obesity, weight loss

## Abstract

***Background:*** Obesity prevalence is higher in women veterans overall than their civilian counterparts considering 44% of women veterans are obese. Thus, there is a critical need to understand the facilitators and barriers to women veterans' participation in weight management programs. The objective of this study is to explore facilitators and barriers to weight loss for women veterans enrolled in the Veterans Health Administration Motivating Overweight/Obese Veterans Everywhere (VA MOVE!) weight management program and gather feedback on the design and delivery of the MOVE! program.

***Materials and Methods:*** Primary qualitative data were collected from women veterans who completed at least one MOVE! visit *via* semistructured telephone interviews. Two authors independently reviewed transcripts for data-derived codes. A content analysis approach was used within the software to code the transcripts.

***Results:*** The mean age of participants was 52 years. Sixty-eight percent (*N* = 17/25) were black, and 52% (*N* = 13/25) lived >64 kilometers from the location of the MOVE! program. Facilitators to participation included both intrinsic (*e.g.*, drive to become healthy) and extrinsic (*e.g.*, drive to improve laboratories) motivating factors. Women expressed difficulty with learning in a group setting and applying lessons to their everyday lives. Others reported the setup of group classes triggered their post-traumatic stress disorder and prevented them from fully participating in the program. Additional barriers included distance traveled to group sessions and lack of access to exercise space.

***Conclusions:*** Our results illuminate barriers and facilitators to engagement in the MOVE! program. Many of the barriers highlighted by these women veterans mirror barriers civilian women face, highlighting the possibility that our results could be applied to other programs designed to target weight loss in women.

## Introduction

Obesity is associated with the leading causes of preventable death in the United States including cardiovascular disease, stroke, type 2 diabetes, and certain types of cancer.^1^ An alarming 41% of all adult women are obese and >50% of non-Hispanic black and Hispanic women are obese.^1^ Obesity prevalence is higher in women veterans overall than their civilian counterparts considering 44% of women veterans are obese.^2^ Weight gain throughout the life cycle is also a considerable problem and data show that mean body weight among women veterans increased from 2000 to 2014.^3^

Although the military places an emphasis on fitness and maintaining a healthy weight, research demonstrates veterans have a propensity to be overweight or of obese status after military discharge due to significant changes in eating habits, physical activity, and psychological comorbidity.^4^ A study of veteran's weight trajectory demonstrated women who left the military during the time of follow-up gained an average of 6.3 kg within a 6-year period compared with 4 kg in women who continued their military service. Furthermore, the mean annual weight gain rates were two times greater before discharge and at the time of discharge than during service or after discharge.^5^

Weight loss has the potential to mitigate complications associated with overweight and obesity. Behavioral interventions are an effective strategy for weight loss. However, prior studies of the Veterans Health Administration Motivating Overweight/Obese Veterans Everywhere (MOVE!) weight management program, an evidenced multidisciplinary approach to weight management, demonstrate that most women enrolled in MOVE! did not achieve clinically significant weight loss (>5% loss).^6,7^ Specifically, the observed mean percentage change in weight for women was −1.5% (standard deviation 5.2).^8^

Additional data from a comparative study of the Aspiring for Lifelong Health (ASPIRE-VA) trial provides additional insight regarding weight loss in men and women veterans. The ASPIRE-VA trial was a randomized controlled trial that evaluated whether a “small change” approach to weight loss using group visits or telephonic coaching could lead to greater weight loss in men and women veterans than the MOVE! weight loss program. The results showed that women participants had poor short-term weight loss in all three groups (ASPIRE group visits, ASPIRE—phone groups and traditional MOVE!). After 12 months, there was <5% weight loss in the “in-person” groups (ASPIRE-Group, −2.6%, MOVE! 2.7%) and an overall weight gain in the “telephone only” arm (ASPIRE-Phone, +0.2%).^9^

Thus, there remains a critical need for a better understanding of the facilitators and barriers to women veterans' participation in weight management programs. This need for better understanding is especially true considering that research shows that women veterans are more likely to enroll in VA-sponsored lifestyle programs than men and endorse high levels of self-management support.^10^

Prior research theorizes that lack of social support, high childcare burden, post-traumatic stress disorder (PTSD), and depression are possible barriers to weight loss for women veterans.^9^ Similar barriers exist for women in the general population.^11,12^ An additional barrier that may be unique to women veterans includes difficulty with attending mixed gender groups because of exposure to military sexual trauma (MST). Nearly one-quarter of women veterans experienced MST and as a result often express preferences for mental health services and primary care delivery in settings that are specifically designated for women.^13^ However, prior studies have not found a preference for women-specific care delivery of management services.^9,13^

Designing weight management strategies for special populations requires an understanding of the desires and needs of the target population. Few studies to date have explored the experience of women veterans in weight loss studies or barriers women face during their weight loss journey. One qualitative study conducted by Moin et al. specifically focused on a web-based diabetes prevention program (DPP) intervention.^14^ Although the results provided valuable lessons regarding the experience of women veterans in the web-based intervention, the study does not address the experience of women in the MOVE! program.

The objective of this analysis was to explore facilitators and barriers to weight loss for women veterans who participated in MOVE! program and gather feedback on the design and delivery of the MOVE! program. The goal of this analysis is to report on lessons learned during semistructured interviews with women veterans. The local VA IRB approved all aspects of the study.

## Materials and Methods

### MOVE! program

The MOVE! program is an evidence-based lifestyle program that provides counseling on changing lifestyle to increase physical activity and improve nutrition. Patients qualify for the program if they have a body mass index (BMI) ≥30 kg/m^2^ or a BMI of 25–30 kg/m^2^ with one or more obesity-related conditions listed in the electronic medical record (*e.g.*, diabetes, hypertension, dyslipidemia, sleep apnea, metabolic syndrome, or arthritis). Participants had an option to participate in in-person group visits or telephonic coaching visits for 10–16 weeks depending on the year they enrolled and the mode of delivery. The curriculum covered information on nutrition, physical activity, and stress management. Participants who attended group visits had access to physical activity and cooking demonstrations during the sessions. It should be noted that although the core curriculum provided to MOVE! sites across the country during the time under study was standard, the mode implementation of the program (in-person vs. telephone visits) as well as length (8–16 weeks) varied across sites.

### Participants

The sample consisted of 25 women veterans who receive care at a large academically affiliated VA hospital in the southeast. Inclusion criteria included female gender, age ≥18 years, and participation in at least one MOVE! weight management program visit between 2008 and 2013.

Exclusion criteria for participating in the qualitative interviews included active alcohol/substance abuse, dementia, cancer undergoing active treatment, not having access to a telephone, and an inability to speak English. Exclusion criteria were confirmed by query data from the local MOVE! database and VA data warehouses.

### Recruitment

Eligible women received a recruitment letter by mail that included details about the study. A contact number was provided so potential participants could indicate their interest in the study or opt out of further communication. Participants were contacted by phone for a screening process. During the screen, potential participants were asked to schedule a time for an interview. If a participant declined to be interviewed, she was not contacted again. A total of 27 women were contacted for the study. Two were excluded based on eligibility criteria. One individual declined participation after going through the consent process and the other was deemed ineligible because she had not completed a MOVE! visit. All interviews were conducted by the study principle investigator (PI) (B.C.B.). All participants who completed an interview received $35.

### Materials

#### Semistructured interview guide

A semistructured interview guide was constructed to explore women veterans' experience with the VA MOVE! weight management program. There were 20 questions that covered motivation to participate, experience with aspects of the MOVE! program design, and individual barriers and facilitators of weight loss. The guide is presented in Table 1.

**Table 1.** Semistructured Participant Interview Guide

Opening question	Follow-up questions
What motivated you to join the MOVE! program?	• How long has it been since you were in the program?
	• How did you find out about it?
	• What did you know about the program before you started?
	• What was it like for you to enroll?
What was it like for you to manage your weight before being in the program?	• What made it hard for you?
	• What helped you?
Please tell me whether there have been any changes in your weight or weight management since you've been in MOVE!	• What about stress, depression, PTSD?
Did you sign up to do the program in-person or over the phone?	• Please tell me how you chose (groups vs. phone vs. IVR)?
	• What did you like about doing it this way?
	• What did you not like?
	• How was it for you to be in a group with male veterans?
Overall, was the MOVE! program helpful or not?	• Please explain why or why not.
What did you think about?	• The frequency of the visits
	• The material covered
	• The length of the visits
	• The length of time between the visits
How should the program be changed for you and other women veterans?	• How could the program be changed to help women like you complete the program?
	• How could the program recruit more women to participate?
Is there anything that you would like to mention that I didn't ask about?	

PTSD, post-traumatic stress disorder.

#### Data collection

After the consent process, participants were asked questions from a brief demographic survey that included questions about age, race, education level achieved, and family status, including marital status and number of people living in the home. Presence of a diagnosis of PTSD and mode of participation in MOVE! (in-person vs. MOVE telehealth vs. MOVE telephone lifestyle coaching [TLC]) were gathered through chart review.

The interviews were conducted in the fall of 2015 by telephone and lasted on average for 50 minutes (range = 40–90 minutes). Interviews were audiotaped and transcribed verbatim by a study research assistant and saved under a unique study ID number. The interviewer, B.C.B., reviewed all transcripts to ensure there were no major transcription errors or large sections of text missing or uninterpretable.

#### Data analysis

Two researchers (B.C.B. and C.S.B.) used ATLAS ti software (version 7; 2012) to manage and code the qualitative data. A content analysis approach was used within the software to code the transcripts.^15^ To accomplish this content analysis, B.C.B. and C.S.B. independently read the first 10 transcripts and applied individual initial codes (*i.e.*, labels to describe content of textual segments). A discussion of their individually coded 10 transcripts led the researchers to agree on which of the 304 initial codes were of higher and lower order to either be parent codes or subcodes.^16^ They agreed to use the following seven parent codes: motivation, exercise, weight loss, flexibility, PTSD, logistics, and nutrition.

The reviewers applied the revised coding scheme to the first 10 transcripts and to an additional 5 transcripts. Any potential coding discrepancies were discussed and thereafter agreed they had reached thematic saturation. They then compared and analyzed the coded data by using sematic linkages (visual representation of the relationships between parent, subcodes, and quotations) constructed within Atlas ti.7 to further interpret how individual responses to different questions overlapped into varying categories (*i.e.*, within interviews) and also compared the answers provided by participants (*i.e.*, between interviews) to generate common themes related to facilitators of participation, barriers to participation, and feedback on program design. Quotations representative of the common themes were identified.

## Results

Twenty-five telephone interviews were completed. The mean age of participants was 52 years. Sixty-eight percent (17/25) self-identified as black and 4% (1/25) were Hispanic. Sixty-four percent had a diagnosis of PTSD. Thirty-two percent (8/25) were married, 32% (8/25) had an annual household income of <$30,000, and 52% (*N* = 13/25) lived >40 miles from the MOVE! program (Table 2). Eighty-eight percent (22/25) of participants attended in-person MOVE! visits. Eight percent (2/25) participated in MOVE-Telehealth. One person participated in MOVE TLC. All participants reported the enrollment process for MOVE! was easy to navigate, the materials were easy to understand, and the length of group classes and frequency of classes were appropriate.

**Table 2.** Baseline Characteristics Overall and by Gender

Baseline characteristics	Overall (n = 25)
Age, mean (SD)	52.6 (8.5)
Black	17 (68)
Ethnicity	
Hispanic	1 (4)
Not Hispanic	22 (88)
Refused/unknown	2 (8)
PTSD	16(64)
Marital status	
Married	8 (32)
Distance from VA MOVE site (miles)	
0–20	8 (32)
21–40	3 (12)
41–60	8 (32)
61–80	4 (16)
81–100	1 (4%)
101 or more miles	0
Missing	1 (4%)
Household income	
$10,000–$19,000	2 (8%)
$20,000–$29,000	6 (24%)
$30,000–$39,000	3 (12%)
$40,000–$49,000	1 (4%)
$50,000–$59,000	3 (12%)
$60,000–$79,000	1 (4%)
$80,000 or more	3 (12%)
Missing	6 (24%)
Highest education level	
High school or GED	2 (8)
Trade/technical/vocational	0 (0)
Some college credit	6 (24)
Associates degree (AA or AS)	11 (44)
Bachelor's degree (BA or BS)	4 (16)
Postgraduate/graduate degree	2 (8)
Job status	
Full time	4 (16)
Part time	4 (16)
Unemployed	2 (8)
Retired	1 (4)
Disabled	14 (56)

Unless otherwise specified, no. (%) with characteristic shown.

MOVE, Motivating Overweight/Obese Veterans Everywhere; SD, standard deviation.

Three major themes from the conversations with women veterans emerged and are discussed in greater detail hereunder.

### Facilitators of participation in the MOVE! program

Participants described factors that motivated them to participate in the MOVE! program. These motivating factors were either intrinsic (driven by internal reward) or extrinsic (driven by external reward). An example of an intrinsic motivator was to lose weight to improve health and ability to participate in family activities. An example of an extrinsic motivator was to achieve a specific health goal (*e.g.*, improve abnormal laboratories). One participant noted, “I went for a doctor appointment and my cholesterol was kind of high… I wanted to avoid the medication; so, I wanted to do what I needed to do to be able to get it down without having to take medication.” An extrinsic motivator included sharing a common goal with other participants. One participant described her relationship with her friend and said, “Well we were… trying to lose weight. So, it was basically just the inspiration or motivation of another veteran trying to do the same thing that I wanted to do.” Another participant indicated that the group itself provided motivation for her to lose weight. She said, “…having a support group is part of getting motivated because they are there to help you stay focused and to also get you back on the band wagon if you fall off. I'll watch out for you, you watch out for me, you know that kind of scenario.” Those who said that external sources motivated them indicated that they had greater success in the beginning of the program, when they were going regularly to group classes, than when they attended fewer classes.

### Barriers to participation and weight loss

Participants shared barriers to participation in the program as well as to weight loss during the program. Many women reported that distance to travel to the sessions and difficulty parking were barriers to participation. In addition, one participant offered that the classroom lecture style of learning as a group prevented her from fully engaging in the program: She said, “That's boring. I mean I was [there] for weight loss and [they] were just talking about how to eat and counting calories. You need something a little bit more than that. Something to help motivate [me] a little bit more…the education piece is important still but…I'm sitting in the classroom the whole time.”

Other women noted the location and setup of group sessions were a trigger for their PTSD. For example, one participant revealed that being in a small noisy room made it difficult to participate in the group session she attended:

I don't do large groups. I don't like being in a crowd… and we were in a small room. Small for me… you got to do your homework with a veteran that has PTSD. You cannot just throw them in a sardine room with a bunch of people.

Women described having difficulty with weight loss due to factors affecting both their nutritional habits and physical activity. One participant discussed her lack of knowledge about nutrition basics. She explained that she learned about nutrition while in the military but described a lack of understanding of the “management of nutrition.” Others opened up about how PTSD is associated with overeating and bingeing. One participant noted,

I have PTSD, I'm also diagnosed as Bipolar, some yeah I'm a stress eater, big time. If I get upset, I eat… Eating is my comfort. It's my security blanket. And even though it may be I'm eating the wrong things. It's easy for me; one of the worse things about my PTSD is the fact that I really can in my mind switch things to the point where it's unreasonable, I know it's illogical to stress out on stuff. But I can't do anything about it short of putting something in my mouth it seems like.

Lastly, participants noted structural barriers (lack of VA exercise space or affordable community-based options) limited their ability to increase physical activity. Coexisting physical health barriers, like asthma, also reduced many women's ability to be physically active.

### Feedback on program design

Participants gave feedback on the process of enrollment in the program, timing of the in-person classes, design of the materials, the method of delivery of in-person group sessions, and ability to translate what was learned to everyday life. Many participants noted it was easy to enroll in the program and felt the timing of classes (length and frequency) was appropriate. One participant noted the design of the materials regarding caloric intake was lacking because it only provided information for men and lacked information regarding goals for women. Another noted that it was difficult to translate what she learned in the classroom setting about nutrition when shopping or meal planning. Specifically, a participant stated, “… ‘how can I apply this?’ was the main issue to me….” Yet another participant noted the need for more one-on-one time with the MOVE! coordinators, which could allow for a more personal discussion—something that is not available during the group setting. Another volunteered that a food list to help with shopping would have been useful. Additional feedback on recipes included the observation that recipes should include low-cost ingredients or foods that the individual likes to eat. One participant noted the recipes were “different than I normally eat and some ingredients were something I would probably would never use but that one time… [it is] not cost wise.”

Lastly, providing personalized workouts was specifically highlighted by another participant,

I'm thinking if it's a way to… give them alternatives like you know you might not be able to go walking like this but this is what you could do you could do a little walk program on TV or you could just exercise fifteen minutes and work your way up to it.

Whether single gender groups should be available was raised by most participants, but the importance of gender separation was not universally agreed upon. One participant specifically focused on lack of single gender groups:

I didn't feel comfortable in the place with a lot of men… it's a unique thing that women are challenged with like they probably couldn't talk about (when) the men are there.

Positives regarding the program design were also noted. One participant communicated that the staff provided some feedback and flexibility regarding physical activity: “They do really try to give you alternate plan of how to do physical exercise when you're not able to do the routine exercise everyone else can do.” Other participants communicated that the number of group visits as well as the timing of visits worked well for them.

## Discussion

The MOVE! program is one of the largest behavioral lifestyle interventions implemented in the United States. Prior studies of MOVE! highlight factors associated with greater weight loss (*i.e.*, standard curriculum and higher participant engagement).^17,18^ However, weight loss for mixed gender groups demonstrates that only 18.7% of participants between 2004 and 2014 achieved clinically significant weight loss after 12 months in the program.^17^ Similarly, an analysis evaluating weight loss for women and men separately demonstrates less than half of men and women achieve clinically significant weight loss (20.4% of women and 21.4% of men lost ≥5% of their weight).^8^ Understanding the experience of MOVE! participants is critical to improving rates of weight loss achieved. The results of this analysis provide important insight into the experience of women veterans who participated in MOVE!

Our results highlight that women were driven by intrinsic and extrinsic factors motivating factors to join the MOVE! program. Participants noted positive aspects to the program including ease of enrollment and appropriate length of classes. Intrinsic and extrinsic motivators to lose weight were noted by all. A common barrier included difficulty with translating lessons learned into actionable steps to reach goals.

Another key barrier included logistic concerns specific to distance from sites where group visits were held, difficulty with parking. When removed, these barriers can lead to greater weight loss than achieved in the traditional in-person MOVE! program. For example, Rutledge et al. investigated differences in weight loss for participants in the MOVE! program as compared with the TeleMOVE program (home program utilizing telehealth unit and scale). Traditional MOVE! participants showed significantly less weight loss as compared with participants in the TeleMOVE program (mean weight loss 1.8% vs. 3.6%, respectively).^19^

Another challenge highlighted by women was barriers associated with the diagnosis of PTSD. The feedback we gathered regarding the role of PTSD as a barrier to participation is noteworthy considering prior research by Buta et al. demonstrating that women in the postdeployment period who had a diagnosis of PTSD had higher BMI growth rates than women without a diagnosis of PTSD (0.11 kg/m^2^; *p* < 0.001).^20^ There remains a lack of understanding regarding how to best adapt interventions for women veterans with PTSD.

Our findings stand in contrast to a qualitative analysis that included women veterans enrolled in a DPP. Women enrolled in the web-based program noted the on-line program was difficult to figure out. In contrast, our participants did not find the MOVE! materials difficult to navigate; however, respondents did report that it was difficult to incorporate MOVE! lessons into daily life and noted many logistical concerns regarding the in-person format.

Another distinction between our results and the web-based DPP involves participant perceptions about the personal nature of the programs. Participants in the web-based DPP described the program as impersonal. The MOVE! program was not deemed impersonal—many participants shared that the group visits—and the personal contact with other participants—kept them motivated.

Results of a secondary analysis of data collected from the Aspire-VA trial by Vimalananda et al. also observed interesting findings regarding weight loss for women enrolled.^9^ They found that women in the Aspire phone group did not lose weight despite high levels of engagement and satisfaction. Our results and those of Vimalananda et al. highlight the challenges that exist in trying to adapt interventions to target weight loss in special populations.

It is notable that in the time since the interviews were conducted, multiple MOVE! sites have tried to address some of the major barriers to participation in MOVE! by offering telephonic coaching, hands-on cooking demonstrations, in-person physical activity components, and access to mobile and texting applications. However, the in-person sessions remain the most popular way to engage in the program.

Additional research is needed to determine how to adapt the program to match the needs of individual participants and utilize telehealth and text messaging to facilitate delivery and enhance effectiveness of the program. In addition, inclusion of mental health components that specifically address the role PTSD and trauma play in weight gain may be particularly effective for women and men who endorse either condition.

The limitations of our study include small sample size and limited generalizability of the results to the civilian population or the veteran population that does not consistently use the VA for their care or are dual users (utilize VA and non-VA services). The strengths of this analysis are inclusion of women who were directly engaged in the program, the broad nature of questions, and the ethnic/racial diversity of the sample.

## Conclusions

Our results illuminate barriers and facilitators to engagement in the MOVE! program. Many of the barriers highlighted by women veterans mirror barriers civilian women face, highlighting the possibility that our results could be applied to other programs designed to target weight loss in women.

## Disclaimer

The content of this article is solely the responsibility of the authors and does not necessarily reflect the position or policy of Duke University, the U.S. Department of Veterans Affairs, the National or the U.S. government. The Department of Veterans Affairs and the National Center for Advancing Translational Sciences of the National Institutes of Health had no role in the design, analysis, or writing of this article.

## Abbreviations Used

ASPIRE-VA: Aspiring for Lifelong Health
BMI: body mass index
MOVE: Motivating Overweight/Obese Veterans Everywhere
MST: military sexual trauma
PTSD: post-traumatic stress disorder
SD: standard deviation
TLC: telephone lifestyle coaching
